# Simulating hierarchical data to assess the utility of ecological versus multilevel analyses in obtaining individual-level causal effects

**DOI:** 10.1186/s12874-025-02504-6

**Published:** 2025-03-22

**Authors:** Lydia Kakampakou, Jonathan Stokes, Andreas Hoehn, Marc de Kamps, Wiktoria Lawniczak, Kellyn F. Arnold, Elizabeth M. A. Hensor, Alison J. Heppenstall, Mark S. Gilthorpe

**Affiliations:** 1 Department of Mathematics and Statistics, Lancaster University, Fylde College, Lancaster, LA1 4YF UK; 2 MRC/CSO Social and Public Health Sciences Unit, School of Health and Wellbeing, University of Glasgow, Clarice Pears Building, 90 Byres Road, Glasgow, G12 8TB UK; 3 School of Computing, University of Leeds, Leeds, LS2 9JT UK; 4 IQVIA, The Point, 37 North Wharf Road, London, W2 1AF UK; 5 Leeds Institute of Rheumatic and Musculoskeletal Medicine, School of Medicine, University of Leeds, & NIHR Leeds Biomedical Research Centre, Chapel Allerton Hospital, Chapeltown Road, Leeds, LS7 4SA UK; 6 School of Social & Political Sciences, University of Glasgow, Adam Smith Building, Bute Gardens, Glasgow, G12 8RT UK; 7 Obesity Institute, Leeds Beckett University, Headingley Campus, Leeds, LS6 3QS UK

**Keywords:** Causal inference, Hierarchical simulations, Multilevel modelling, Ecological analyses, Directed acyclic graphs, Agent-based modelling, Ecological fallacy, Modifiable areal unit problem, Aggregations bias

## Abstract

Understanding causality, over mere association, is vital for researchers wishing to inform policy and decision making – for example, when seeking to improve population health outcomes. Yet, contemporary causal inference methods have not fully tackled the complexity of data hierarchies, such as the clustering of people within households, neighbourhoods, cities, or regions. However, complex data hierarchies are the rule rather than the exception. Gaining an understanding of these hierarchies is important for complex population outcomes, such as non-communicable disease, which is impacted by various social determinants at different levels of the data hierarchy. The alternative of analysing aggregated data could introduce well-known biases, such as the ecological fallacy or the modifiable areal unit problem. We devise a hierarchical causal diagram that encodes the multilevel data generating mechanism anticipated when evaluating non-communicable diseases in a population. The causal diagram informs data simulation. We also provide a flexible tool to generate synthetic population data that captures all multilevel causal structures, including a cross-level effect due to cluster size. For the very first time, we can then quantify the ecological fallacy within a formal causal framework to show that individual-level data are essential to assess causal relationships that affect the individual. This study also illustrates the importance of causally structured synthetic data for use with other methods, such as Agent Based Modelling or Microsimulation Modelling. Many methodological challenges remain for robust causal evaluation of multilevel data, but this study provides a foundation to investigate these.

## Introduction

Many countries have a long history of collecting data on the demographics, socioeconomic profiles, health characteristics, and service use of their populations. For example, many of the contemporary population-based registers in the Nordic countries date back to the 1960s and 1970s [1]. Purposes of collecting data on micro units, such as households and individuals, can be manyfold and range from descriptive monitoring for administration or resource allocation, to evaluating or predicting outcomes of different policies and interventions [1]. More recently, with the advent of ‘big data’, large enterprises have also started to collate and study large-scale population datasets. Similarly, their aims tend to relate ultimately to micro unit outcomes (e.g., the customer base), monitoring, evaluating, or making predictions related to new processes and innovations [2].

Even though causal effect estimation is typically sought as an ‘average treatment effect’ (ATE) for a target population of interest, understanding of the causal mechanism operating often lies at the micro unit – especially when the focus is around health outcomes, as these are intrinsically individual-level experiences. To do so necessitates individual-level simulated data to examine how causal processes relate to the individual experience. Although some study designs seeking causal insights do not require individual-level data – e.g., econometric methods, such as difference-in-difference – these rely on the rollout of interventions in terms of timing, geographical spread, and other assumptions that may not always be met, and simulations of aggregated single-level data are then sufficient to validate these methods. However, interest often extends to understanding population inequalities, where individual-level data are then required. These other methods therefore do not overcome the need for individual synthetic data if the assessment of the ecological phenomenon extends to understanding consequences at the individual level.

In many real-world situations, the data required are therefore typically hierarchical. For example, data on primary healthcare use are structured with individuals grouped by their general practitioner (GP), which are grouped by practice, grouped by administrative health boards, and grouped within a national context. Boundaries and cluster sizes are derived for administrative convenience, e.g., being geographically coherent and/or historically retaining focus on established sub populations. Most population-level interventions, e.g., practice-level changes, or national-level primary care policies, may be implemented across population higher levels due to practical reasons of implementation at scale, yet insightful impacts are sought at the micro unit, i.e., aimed at understanding the causal relationships among individuals.

It is recognised that there are often interactions between individuals, within and between clusters, as well as between individuals and clusters [3]. Disentangling these relationships is of considerable interest to population researchers. Inherent hierarchical structures create a range of analytical challenges. Consequently, methods that work well for a single homogeneous population may lead to misleading results if applied naively to a population with a substructure that is not acknowledged explicitly. It is therefore necessary to account for such substructures when analysing population data. For instance, a health study in Scotland used *mixed effects modelling* (also known as *multilevel modelling* [4]) to disentangle the heterogeneity in prescribing behaviours among GPs [5]. By modelling the data hierarchy of GPs nested within practices the study explored if a “high-risk-prescribing culture” was driven by idiosyncrasies of individual GPs or by practice-level culture. It was found that high-risk prescribing was more of an individual-GP issue than a practice-level phenomenon [5], an insight which might subsequently inform possible interventions.

Multilevel modelling is a group of powerful data analytic techniques that explore variables at all levels of multiple hierarchies simultaneously to evaluate associations among individuals and population clusters. Multilevel modelling is ubiquitous across many fields of social and health research [6–8]. However, unless deployed explicitly to yield insights into cause and effect, these methods reveal correlational relationships. In many forms of data analysis, the question of analysing cause and effect is of central importance, yet correlation does not mean causation. If the goal is to inform *interventions*, a causal understanding is essential [9]. If $X$ does not cause $Y$ but is only associated with it, an intervention on $X$ would be the wrong decision if, ultimately, $Y$ is the target outcome of the intervention.

Although a multilevel analysis of Scottish GP prescribing data was informative in estimating the relative strength of associations at one level of the hierarchy versus another, this could not unpick what was operating *causally*, and it could not quantify any effect sizes of such causes. To understand causal implications, we must perform multilevel modelling within a formal causal framework and view the results through a causal lens. Otherwise, cause and effect might be confused, with grave consequences, leading to potential implementation of ineffective or even harmful policies, wasting valuable resources that could otherwise be more effectively allocated. Instead of informing ‘possible intervention approaches’ from correlational studies, a causal lens should inform the most (cost-)effective intervention.

Although causal inquiry is viewed by some as the sole preserve of randomised control trials (RCTs), many RCTs are impossible to conduct (ethically or practically), and observational research must instead yield causal insights. Observational research does not automatically convey causal insights, which may only be appropriately interpreted as causal if robust causal inference methods have been used. To date, the application of contemporary causal inference methods has not been a signature of most ongoing observational research [9], with vague and considerably less robust ‘risk factor’ correlational approaches prevailing [10]. Observational causal research is not as analytically straightforward as an RCT. Identifying and quantifying the wider social and environmental determinants of health involves asking causal questions of individuals who inhabit a complex real-world system with multiple inherent hierarchies.

Modelling causal structures has been sought in varied ways. One of the more sophisticated strategies is agent-based modelling (ABM) [11] – a simulation comprising individual ‘agents’ that are modelled using sequential stochastic processes to emulate real-time transitions in life. This embraces complexity to the point that emergent properties may appear that are not discoverable using simpler strategies. ABMs must, however, be parameterised to reflect true underlying (causal) data generating mechanisms (DGMs) at all levels of a data hierarchy, which demands *a priori* understanding of how the data structures come into being [12]. If ABMs are used with synthetic data, such data must capture all causal structures known or postulated as likely for the models to elicit causal insights – currently this is not common practice.

It is also essential to understand a system’s DGM to inform which of a plethora of analytical strategies available is the most appropriate [13]. While contemporary causal inference methods are based on potential outcomes [14] and counterfactuals [15], these can involve graphical model theory [16], which has spawned the use of *directed acyclic graphs* (DAGs) [17, 18] to encode all causal assumptions made of the DGM for an observed system. Causal methods developed to date predominantly treat data as ‘flat’, i.e., non-hierarchical. It is therefore important to extend causal methods and causal diagrams to operate with hierarchical data.

Contemporary causal inference methods have not fully tackled the complexities of data hierarchies, apart from repeated measures in longitudinal data, where developments include the evaluation of time-varying outcomes, time-varying exposures, and time-varying confounders, using any of the three g-methods [19], or variations thereof [20, 21]. At the same time, causal inference methods have become increasingly popular in demographic research, where g-methods have made an important contribution as part of studies with a designated lifecourse approach. Examples are diverse and have covered different elements of the lifecourse ranging from the socioeconomic determinants of fertility [22] to retirement and cognitive functioning [23]. Longitudinal data may also be subject to causal mediation analysis [24], where the causal impact of an exposure is evaluated for its *direct* impact on an outcome, separately from its influences *via* one or more mediators – i.e., intermediate variables that lie on the causal path between exposure and outcome. Although longitudinal data are hierarchical, developments in causal inference methods have not focused on data hierarchy per se, leaving a gap in our capabilities to interrogate complex hierarchical data.

There is also an entire discipline concerned with interactions between individuals within clusters that does not exploit the full data hierarchy – *ecological analyses* [25] – but these methods suffer an issue of widespread interest within population research surrounding the role of cluster size, described as the *modifiable areal unit problem* (MAUP) [26]. MAUP is where different results emerge according to the size of clusters used for the same dataset. This issue plagues many aspects of population spatial analyses [27–29], and affects the robustness of research in epidemiology [30–32] and economics [33–35]. It is important to understand these differences from a causal perspective. While this is not the focus of this study, what is proposed provides the means to explore MAUP.

DAGs are often used to inform decisions about which variables to control for (referred to as confounders) and which not (e.g., mediators) – the latter running the risk of ‘Table 2 Fallacy’ [36] and other inferential biases including reversal paradox [37] and collider bias [38]. Being a cause of both exposure and outcome is not sufficient to be classified as a confounder since complex confounding situations can arise such that what looks to be genuine confounding, if adjusted for, introduces more bias (e.g., M-Bias; [38, 39]), which is why we use graphical model theory that underpins DAGs to determine all true confounding [40]. Simulating data with the aid of a DAG and comparing the resulting covariance structure with what is observed in real data turns out to be surprisingly informative due to the level of implied constraints that are explicitly encoded from external theory, knowledge, or mere supposition [41]. This is because the implied constraints within a DAG can be sufficient to settle some cause-and-effect questions with no need for real data; merely simulating the DGM assumptions can be enlightening. Principles underpinning the development of causal diagrams are discussed in the Appendix (Section "Important principles underpinning the development of causal graphs").

Inspired by MAUP, and a desire to understand the causal implications of cross-level associations related to cluster size, this study explores multilevel and ecological models from a causal viewpoint where a cross-level relationship exists between cluster size and two individual-level variables. We adopt a hierarchical DAG-type approach to gain insights into how causal questions concerning individuals are affected by a cross-level relationship with known causal origins. We first comprehend causal relationships in hierarchical data by describing what intrinsic real-world environmental features influence how hierarchies arise. The development of a *hierarchical causal diagram* that encodes a DGM is essential, from which causally structured hierarchical data may then be simulated – if it is not feasible to simulate data from a causal diagram, that diagram has limited or zero utility.

The causal diagram need not be a formal DAG (i.e., where all nodes are probabilistic and follow mathematical concepts and rules derived to inform robust causal enquiry [42]), providing the diagram can inform meaningful simulation. Our strategy therefore begins by depicting all probabilistic variables at level-1 in a DAG, while variables at level-2 are determined by aggregation [43], placing individuals into clusters in a judicious manner to ensure we maintain all individual-level causal relationships and cross-level associations between cluster size and individual-level variables of interest. We then ask: *Can we robustly estimate via multilevel and/or ecological analyses the population average causal effect for a process that affects individuals?*

This study seeks several novel contributions: 1) how to devise a causal diagram for hierarchical data, cognizant of how to handle deterministic variables in a formal causal framework; 2) outline an algorithm that ensures a cross-level causal relationship is present in the simulated data; 3) quantify the extent of ecological fallacy bias for the first time; and 4) demonstrate *via* simulation how individual-level data are essential for estimating causal effects impacting individuals.

## Methods

Our objectives were three-fold: 1) draw a hierarchical causal diagram to encode a DGM that invokes a cross-level relationship between two individual-level variables and cluster size with prespecified causal origins; 2) simulate lower-level data with the structure depicted in the DGM and derive upper-level data by aggregation; and 3) explore multilevel and ecological analyses of the simulated data to estimate population average causal relationship among individuals, contrasting findings with the simulated truth to identify the presence and extent of *residual confounding bias* (which is due to incomplete confounding adjustment) and *aggregation bias* (which is due to data transformation prior to analysis).

The DGM, simulations, and analyses are summarised in the following steps:

1. Draw the hierarchical causal diagram.
2. Simulate individual-level population data (variables have suffix $i$).
3. Cluster individual-level data in a manner that retains a cross-level relationship with prespecified causal origins.
4. Aggregate individual-level data by clusters (variables have suffix $j$).
5. Undertake multilevel modelling of the full dataset to estimate a population average causal effect for two individual-level variables.
6. Undertake multiple ecological analyses of the aggregated data to estimate a population average causal effect for the same individual-level variables.
7. Contrast the multilevel and ecological causal estimates with the true causal effect simulated to identify the potential and extent of any biases.

### Our data generating mechanism (DGM)

Any number of plausible DGMs could give rise to hierarchically structured data – too many to be evaluated in a single study. Some simplifying restrictions were therefore considered. We limited ourselves to situations in which we anticipate ‘*population homogeneity*’ and ‘*no interference*’ (as termed in the causal inference literature [44]). These restrictions provide an underpinning DGM for which we establish the principles of a hierarchical causal diagram to inform the simulation of causally structured hierarchical data. Despite narrowing our choice of DGM, the assumptions adopted produce generalisable insights for large swathes of population research and other, more specific DGMs can be the focus of future studies.

Population homogeneity describes situations where variable relationships are similar across clusters, and where these relationships are subject to cluster-specific influences that vary but are nevertheless generalisable to a population. This applies to situations in which individuals experience overarching ‘norms’ within a population, with cluster differences between subregions – as within a country, for instance. The alternative, where clusters substantially differ (which could be for myriad reasons) is more complex and is consistent with clusters being separate countries, each with its own socio-political ‘norms’ that yield substantial between-cluster (i.e., between-country) heterogeneity.

The no interference assumption implies that individual-level variable relationships are not affected by the status of other individuals. Within the ABM literature, interference equates to agent-to-agent interactions, as observed with infectious diseases, for instance. The no interference assumption is unrealistic for some situations but is compatible with many real-world scenarios where the potential for network/peer effects are accommodated via the clustering, providing this is modelled explicitly and correctly. No interference is consistent with studying non-communicable diseases in the context of hierarchically structured health data.

**Fig. 1:**
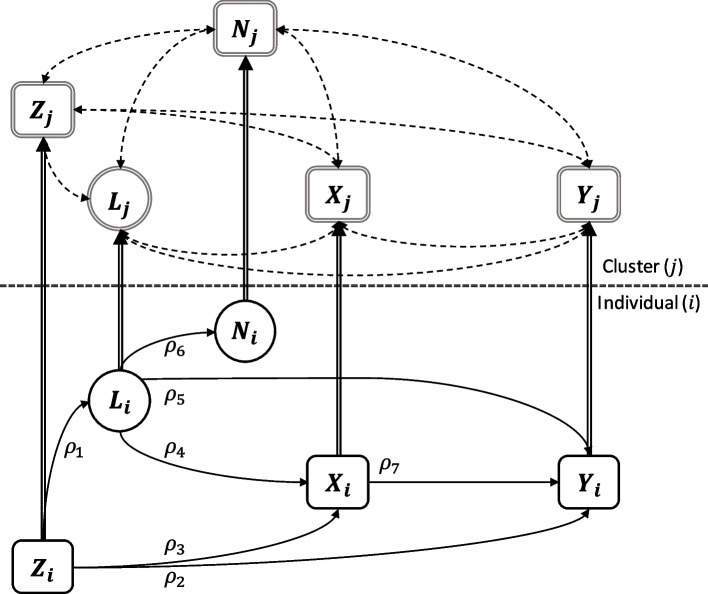
A hierarchical causal diagram illustrates individual-level causal relationships among five variables (circles are unobserved, i.e., latent; squares are observed; double-edged enclosures are determined variables): $Y$, the outcome; $X$, the exposure; $Z$, a ‘regular’ confounder of the $X-Y$ relationship that is observed; $L$, a latent confounder of the $X-Y$ relationship that is unobserved but affects individual-level latent variable ${N}_{i}$, which manifests as an observed cluster-level feature, ${N}_{j}$. The solid single arrows signify causal relationships between variables; dashed lines are bivariate correlations realised among aggregated cluster-level (fully determined) variables; and double-lined arrows indicate deterministic pathways [43]

Figure 1 illustrates a hierarchical causal diagram for the DGM used in this study. We defined a specified causal relationship between individual-level exposure variable ${X}_{i}$ and individual-level outcome variable ${Y}_{i}$. As confounding is a concern in observational studies, this was considered in two forms: *regular* confounding by ${Z}_{i}$, which is a ‘super-variable’ – i.e., a single variable that represents all potential individual-level known and *observed* confounders; and *latent* confounding by ${L}_{i}$, – another ‘super-variable’ representing all potential individual-level known but *unobserved* confounding. Both confounders are aggregated at the cluster-level (${Z}_{j}$ and ${L}_{j}$), but the cluster-level variable ${L}_{j}$ is also unobserved, meaning adjusting for it in real-world data is impossible. Cross-level relationships occur between the cluster-level variable, ${N}_{j}$, and both ${X}_{i}$ and ${Y}_{i}$, due to the a priori specified causal structure, even though the individual-level latent variable, ${N}_{i}$, which is caused by ${L}_{i}$ has no direct causal effect on either ${X}_{i}$ or ${Y}_{i}$.

**Fig. 2:**
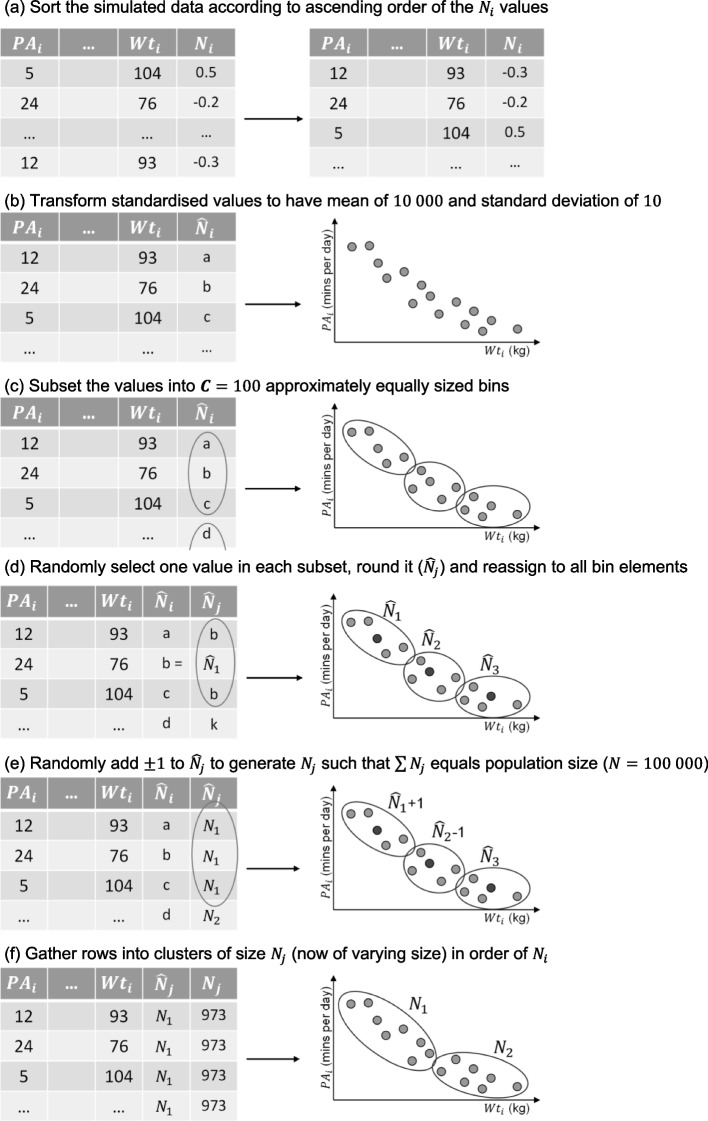
A schematic illustration of the algorithm that transforms an individual-level latent variable into a cluster-level measure of cluster size, which is used to produce the data clusters, illustrated using the example of daily mean levels of physical activity ($PA$) in minutes as the exposure and body weight ($Wt$) in kilograms as the outcome. (footer): The algorithm categorises simulated individual-level data into ${\varvec{C}}$ clusters to convey cross-level associations with causal origins as per the data generating mechanism of Fig. 1. The process involves: (**a**) sorting individual-level data by ascending latent variable ${N}_{i}$ values; (**b**) rescaling such that, once rounded, ${\widehat{N}}_{i}$ are potential cluster sizes with mean $\boldsymbol N/\boldsymbol C=1000$ and standard deviation $10$; (**c**) subset selection into ${\varvec{C}}$ evenly sized subsets – enclosed in the three ellipses; (**d**) randomly select one ${\widehat{N}}_{i}$ value per subset and round to generate ${\varvec{C}}=100$ cluster size values [alternatively, take subgroup means and round]; (**e**) undertake value modification to randomly selected cluster size values by adding or subtracting one to ensure all cluster sizes sum to population size; and (**f**) regroup subsets into unequally sized clusters – enclosed in the two new ellipses – based on the ordered values of ${N}_{i}$

Means were taken of continuous individual-level variables to generate their aggregated cluster-level counterparts, except for ${N}_{j}$, which was derived using the novel algorithm outlined in Fig. 2. Individual-level binary variables were aggregated to count variables. Data thus comprised individual-level variables ${Z}_{i}$, ${L}_{i}$, ${N}_{i}$, ${X}_{i}$, and ${Y}_{i}$ obtained from DAG-informed simulations [42], and derived cluster-level variables ${Z}_{j}$, ${L}_{j}$, ${N}_{j}$, ${X}_{j}$, and ${Y}_{j}$. To acknowledge the deterministic nature of cluster-level variables, we adopt the recommended notation of double-edged enclosures for determined variables and double-lined arcs for deterministic pathways [43].

### Simulations

All individual-level causal relationships (direction and strength) in the hierarchical causal diagram were assigned standardised path coefficients (${\rho }_{1}, \dots ,{\rho }_{7}$), which translate to a Pearson correlation matrix of the individual-level data [45, 46]. The embedded DAG determined a level-1 correlation matrix, but this does not provide information on variable distributions. Where population data comprised only continuous measures, we assumed standardised multivariate normality for individual-level variables, i.e., $\left\{{Z}_{i}{,L}_{i}, {N}_{i}, {X}_{i}, {Y}_{i}\right\}$ ~ $N\left(0,\Sigma \right)$, where $\Sigma$ is the DAG-implied correlation matrix. The ${L}_{i}-{N}_{i}$ causal relationship – giving rise to cross-level associations with specified causal origins – was set to be strong (${\rho }_{6}=0.8$) to minimise residual confounding, i.e., where adjustment for confounding is incomplete, due to using the confounder’s surrogate of cluster size. Estimation of the ${X}_{i}-{Y}_{i}$ causal relationship is expected to be imperfect due to residual confounding bias.

Despite being mathematically convenient, all variables being multivariate normal is unlikely for many contexts. As implications of nonnormal distributions are profound for the outcome – requiring different analytical methods that typically introduce link-function transformations – two main scenarios were considered: 1) all individual-level variables were multivariate normal; and 2) individual-level outcome, ${Y}_{i}$, was binary with a prevalence of $10$%, while all other individual-level variables remained multivariate normal. We additionally explored three adaptations to these scenarios to gain insights into related interesting problems, such as what if: a) the binary outcome had $0.01\%$ prevalence to explore the effect of modelling rare outcomes (e.g., rare diseases) [47], which can create estimation challenges [48]; b) latent confounding was binary ($10$% prevalence) for continuous outcomes; and c) latent confounding was binary for binary outcomes (both with $10$% prevalence).

To limit computational challenges (as simulations take many days) while seeking realism, a population of $\boldsymbol N=100000$ was sought to reflect a country’s subregion (e.g., local authority), and ${\varvec{C}}=100$ small area subclusters (e.g., census geographies). The ${X}_{i}-{Y}_{i}$ standardised path coefficient ${\rho }_{7}$ (i.e., the prespecified standardised causal effect) ranged from $0.0$ to $0.5$ in increments of $0.1$, providing a range of six estimates, a sufficient number to identify various forms of nonlinearity in potential biases. Modest and equal confounding path coefficients of $0.3$ were adopted throughout. Simulations were replicated $1000$ times with model estimates derived as the median of all estimates.

To simulate multivariate normal data we used the R package dagitty [42], which takes a specified DAG to obtain its implied correlation matrix, from which multivariate Normal data are generated. More complex techniques are needed to simulate multivariate data with a mixture of distributions. We used the GenData algorithm in R developed by Ruscio and Kaczetow [49], which benefits from being ‘tuned’ to optimise performance (Appendix, Section "Introduction"). The creation of data with a targeted covariance structure is never guaranteed, as it remains challenging to generate complex multivariate nonnormal data. Simulations were therefore investigated for signs of concern – while some simulations were challenging, there were no concerns with overall findings (Appendix, Section "Introduction"). 

The main simulations thus comprised four scenarios for continuous and binary outcomes:

1. path coefficients were zero except $\rho_{7}$ and $\rho_{2}=\rho_{3}=0.3.$ 
2. path coefficients were zero except $\rho_{7}$ with $\rho_{4}=\rho_{5}=0.3$ and $\rho_{6}=0.8.$
3. path coefficients were nonzero except $\rho_1=0,$ with $\rho_{2}=\rho_{3}=\rho_{4}=\rho_{5}=0.3$ and $\rho_{6}=0.8.$
4. path coefficients were nonzero with $\rho_{1}=0.5,\;\rho_{2}=\rho_{3}=\rho_{4}=\rho_{5}=0.3$ and $\rho_{6}=0.8.$

and three adaptations to Scenario 4:

- 4a) binary outcome prevalence was $0.1$%.
- 4b) ${L}_{i}-$ confounding was binary ($10$% prevalence) while the outcome was continuous.
- 4c) ${L}_{i}-$ confounding and outcome were binary (both $10$% prevalence).

Scenario 1 explored individual-level regular confounding by setting nonzero path coefficients for the ${Z}_{i}-{X}_{i}$ and ${Z}_{i}-{Y}_{i}$ relationships. Ecological analyses accommodated this by adjusting for aggregate variable ${Z}_{j}$. Scenario 2 explored unobserved confounding by setting nonzero path coefficients for relationships ${L}_{i}-{X}_{i}$, ${L}_{i}-{Y}_{i}$, and ${L}_{i}-{N}_{i}$; the latter necessary to create cross-level associations between cluster size ${N}_{j}$ and individual-level variables. Ecological analyses addressed unobserved confounding by adjusting for cluster size as its surrogate measure (necessary since the aggregate variable ${L}_{j}$ was latent). Scenario 3 explored the influences of regular and latent confounding combined by setting nonzero path coefficients for relationships ${Z}_{i}-{X}_{i}$, ${Z}_{i}-{Y}_{i}$, ${L}_{i}-{X}_{i}$, ${L}_{i}-{Y}_{i}$, and ${L}_{i}-{N}_{i}$. This explored if residual confounding due to partial adjustment for ${L}_{i}$ confounding was affected by regular confounding by ${Z}_{i}$ – the level 1 DAG implies this should not happen for individual-level data, but the influences of clustering, adoption of ecological analyses, and use of link transformations for binary outcomes might change this. Scenario 4 was the most complex, with regular confounding by ${Z}_{i}$ also influencing latent confounding by ${L}_{i}$, placing greater emphasis on the suboptimal adjustment for the aggregate surrogate of cluster size. The first extension to Scenario 4 (4a) evaluated how binary outcome models performed for rare outcomes; the second and third extensions (4b and 4c) evaluated the impact of binary latent confounding for continuous and binary outcomes, respectively.

### Analyses

For both continuous and binary individual-level outcomes across the four main scenarios, multilevel and several ecological analyses were explored for different approaches to adjusting for cluster size (Table 1). The most straightforward strategy is to include cluster size (${N}_{j}$) as a linear term within the model, i.e., Models 1 & 2 in Table 1: 1) multilevel analyses adjusted for cluster size ${N}_{j}$; and 2) ecological analyses adjusted for cluster size, ${N}_{j}$. Five additional models were considered and are detailed in Appendix Section "Methods". All models adjusted for regular confounding, with multilevel analyses adjusting for ${Z}_{i}$ and ecological analyses adjusting for ${Z}_{j}$. For all binary outcomes we adopted a Poisson model with log link to facilitate comparisons across models.

**Table 1:** Summary of the different models undertaken. All models were adjusted for regular confounding if present (the ecological analyses adjusted for ${Z}_{j}$, while the multilevel analyses adjusted for ${Z}_{i}$). For the multilevel and ecological analyses, where $Y$ was continuous, models were linear; where $Y$ was binary, models 1-3 and 7 were Poisson (log link) while models 4-6 were linear

Model	Model Formula	Outcome Distribution (Link Function)
**Multilevel**		
1 – cluster-size-adjusted	${Y}_{i}\sim {X}_{i}+{N}_{j}$	Normal (identity) / Poisson (log)
**Ecological**		
2 – cluster-size-adjusted	${Y}_{j}\sim {X}_{j}+{N}_{j}$	Normal (identity) / Poisson (log)
3 – inverse-size-adjusted	${Y}_{j}\sim {X}_{j}+1/{N}_{j}$	Normal (identity) / Poisson (log)
4 – ratios-unadjusted	${Y}_{j}/{N}_{j}\sim {X}_{j}/{N}_{j}$	Normal (identity)
5 – ratios-size-adjusted	${Y}_{j}/{N}_{j}\sim {X}_{j}/{N}_{j}+{N}_{j}$	Normal (identity)
6 – ratios-inverse-size-adjusted	${Y}_{j}/{N}_{j}\sim {X}_{j}/{N}_{j}+1/{N}_{j}$	Normal (identity)
7 – log-size-offset	${Y}_{j}\sim {X}_{j}+offset\left(log{N}_{j}\right)$	Poisson (log)

**Fig. 3:**
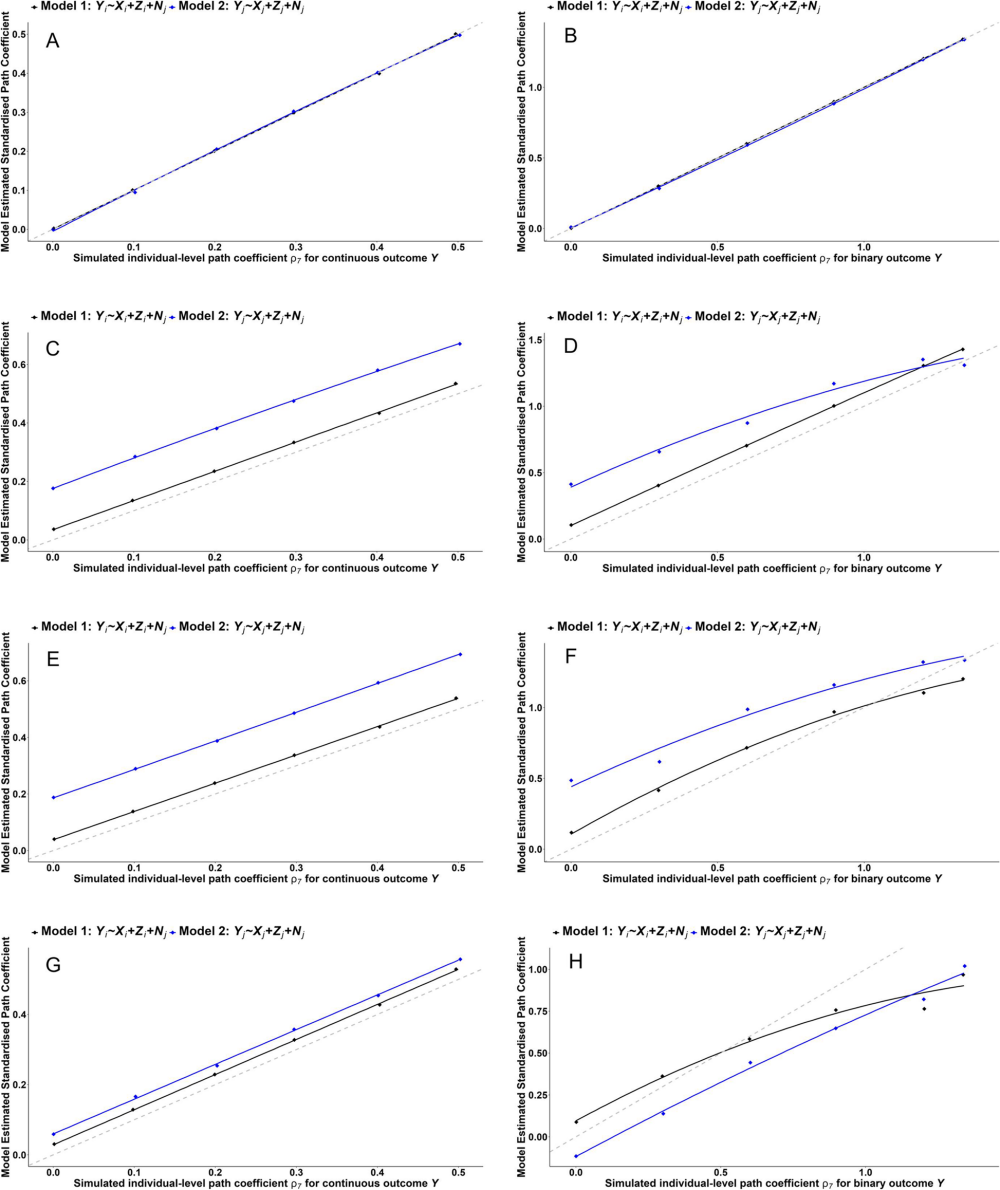
Summary of the multilevel and main ecological analyses of simulated data (plotted in black and blue respectively) for all four scenarios for continuous (charts **A**, **C**, **E, G**) and binary outcomes (charts **B, D,** **F**, **H**) – the diamond shaped plots are median estimates (y-axis) plotted against individual-level simulated ‘true’ effect sizes (x-axis); the dotted grey line indicates perfect agreement between simulated and estimated effect sizes; continuous lines are fitted lines to the median estimates. Scenario 1: Estimates of ${\rho }_{7}$ with *regular* confounding only. Scenario 2: Estimates of ${\rho }_{7}$ with *latent* confounding only. Scenario 3: Estimates of ${\rho }_{7}$ with *regular* and *latent* confounding that *are not* causally related. Scenario 4: Estimates of ${\rho }_{7}$ with *regular* and *latent* confounding that *are* causally related

**Fig. 4:**
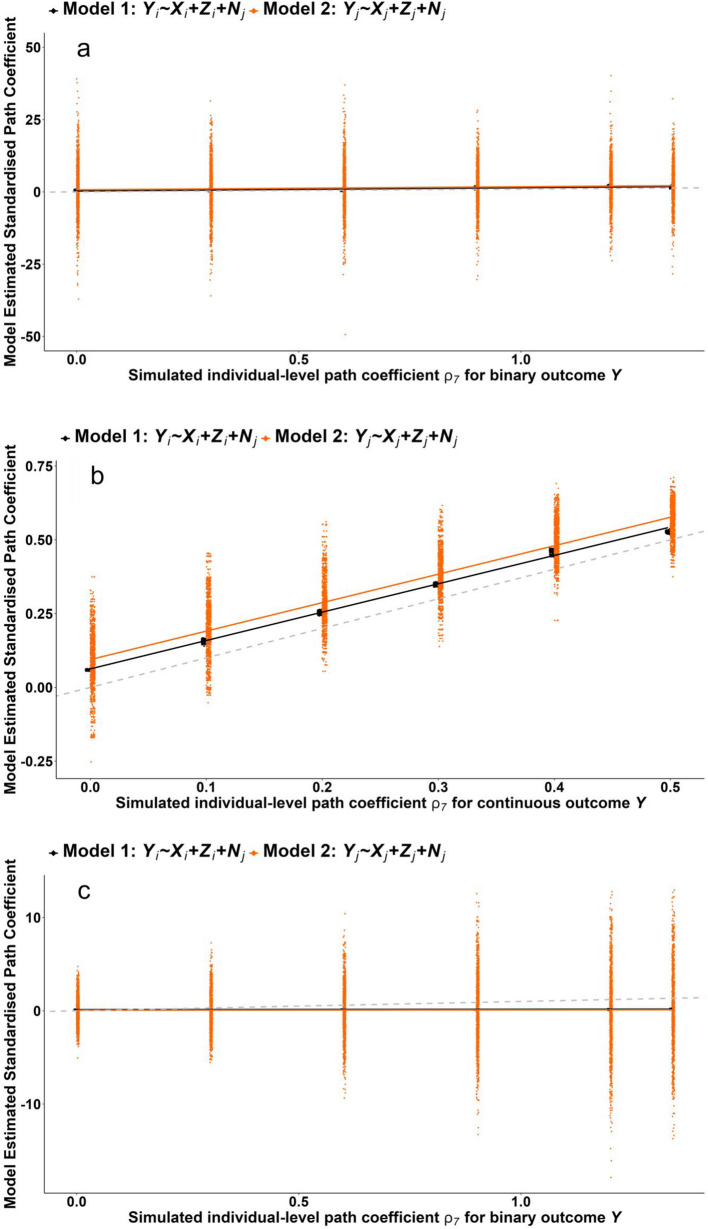
Plots of multilevel and main ecological estimates of simulated data (plotted in black and orange respectively) for Scenario 4 (where estimates of ${\rho }_{7}$ were sought for causally related *regular* and *latent* confounding) with additional complexity considerations: (**a**) low outcome prevalence ($0.1$%); (**b**) binary ${L}_{i}-$ confounding ($10$% prevalence) with continuous outcome; and (**c**) binary ${L}_{i}-$ confounding with binary outcome (both $10$% prevalence). The diamond shaped plots are individual simulation cluster-level estimates (y-axis) plotted against the individual-level simulated ‘true’ effect sizes (x-axis); the grey dotted line depicts perfect agreement between simulated and estimated effect sizes; continuous lines are linear fitted lines to all $1000$ estimates. Scenario 4a: Estimates of ${\rho }_{7}$ with *regular* and *latent* confounding that are causally related with low binary prevalence. Scenario 4b: Estimates of ${\rho }_{7}$ with *regular* and *latent* confounding that are causally related with binary latent confounding and continuous outcome. Scenario 4c: Estimates of ${\rho }_{7}$ with *regular* and *latent* confounding that are causally related with binary latent confounding and binary outcome

We illustrate model results by plotting median estimates for each model against simulated truth (${X}_{i}-{Y}_{i}$ path coefficient ${\rho }_{7}$ in Fig. 1) for the four main scenarios in Fig. 3. For the three extensions to Scenario 4 we plot raw estimates in Fig. 4 to illustrate heterogeneity of model estimates. For visual clarity, charts do not include 95% simulation intervals. For continuous outcomes, true values are the specified path coefficients (i.e., ${\rho }_{7}$); for binary outcomes, true effects must be transformed due to the Poisson log link; and for ecological analyses of ratio outcomes (${Y}_{j}/{N}_{j}$), effect estimates for (${X}_{j}/{N}_{j}$) must be rescaled by dividing through by the number of clusters (Appendix, Section "Methods").

We used the statistical software R version 4.4.1 for all simulations and analyses [50]. The reproducibility pack containing R-code and instuctions on how to replicate or adapt the simulations can be found here: 10.5281/zenodo.14901732.

## Results

Standardised datasets were simulated for the DGM in Fig. 1 for all four main scenarios and three extensions to Scenario 4. Figures 3 and 4 illustrate the results graphically, while Table 2 summarises median and standard deviation of estimated biases. Results show:

**Table 2:** Median and standard deviations (sd) of estimate biases for all scenarios evaluated. Scenarios 1 to 4 consider combinations of continuous regular and latent confounding for continuous and binary ($10\%$ prevalence) outcomes: 1 – regular confounding only; 2 – latent confounding only; 3 – unrelated regular and latent confounding; and 4 – related regular and latent confounding (0.8 path coefficient). Extensions to Scenario 4 include: 4a – low prevalence binary outcomes (0.4%); 4b – binary confounding (10% prevalence) for continuous outcomes; and 4c – binary confounding (10% prevalence) for binary outcomes (10% prevalence)

Scenario	Effect Size	Continuous Outcomes				Effect Size	Binary Outcomes			
		Multilevel Estimates		Ecological Models			Multilevel Estimates		Ecological Models	
		Bias (median)	Uncertainty (sd)	Bias (median)	Uncertainty (sd)		Bias (median)	Uncertainty (sd)	Bias (median)	Uncertainty (sd)
1	0.0	0.000	0.003	−0.003	0.098	0.00	0.000	0.004	0.007	0.300
	0.1	0.000	0.003	−0.006	0.105	0.30	−0.001	0.004	−0.018	0.307
	0.2	0.000	0.003	0.008	0.103	0.60	−0.001	0.008	−0.006	0.286
	0.3	0.000	0.003	0.002	0.091	0.90	−0.001	0.012	−0.016	0.278
	0.4	0.000	0.003	0.001	0.092	1.20	0.003	0.018	−0.005	0.253
	0.5	0.000	0.003	−0.003	0.082	1.33	−0.001	0.019	−0.001	0.255
2	0.0	0.034	0.003	0.174	0.124	0.00	0.103	0.003	0.410	0.959
	0.1	0.035	0.003	0.184	0.124	0.30	0.103	0.005	0.356	1.101
	0.2	0.035	0.003	0.183	0.132	0.60	0.102	0.007	0.276	1.264
	0.3	0.034	0.003	0.175	0.129	0.90	0.104	0.011	0.270	1.474
	0.4	0.034	0.003	0.179	0.126	1.20	0.106	0.017	0.151	1.752
	0.5	0.034	0.003	0.170	0.117	1.33	0.088	0.023	−0.031	1.653
3	0.0	0.038	0.003	0.185	0.128	0.00	0.115	0.005	0.483	0.936
	0.1	0.038	0.003	0.188	0.133	0.30	0.115	0.007	0.316	1.072
	0.2	0.038	0.003	0.190	0.126	0.60	0.115	0.011	0.389	1.334
	0.3	0.038	0.003	0.185	0.127	0.90	0.070	0.015	0.259	1.399
	0.4	0.038	0.003	0.192	0.116	1.20	−0.096	0.017	0.120	1.211
	0.5	0.038	0.002	0.193	0.113	1.33	−0.139	0.019	−0.006	1.075
4	0.0	0.028	0.003	0.057	0.105	0.00	0.086	0.010	−0.118	2.441
	0.1	0.028	0.003	0.065	0.099	0.30	0.062	0.013	−0.163	2.560
	0.2	0.028	0.003	0.056	0.089	0.60	−0.017	0.013	−0.155	2.410
	0.3	0.028	0.002	0.057	0.078	0.90	−0.142	0.013	−0.253	2.128
	0.4	0.028	0.002	0.053	0.067	1.20	−0.435	0.014	−0.380	2.127
	0.5	0.028	0.001	0.057	0.045	1.33	−0.373	0.015	−0.321	1.761
4b / 4a	0.0	0.009	0.001	0.036	0.102	0.00	0.272	0.150	1.011	9.685
	0.1	0.007	0.004	0.050	0.104	0.30	0.041	0.153	0.245	9.462
	0.2	0.004	0.004	0.038	0.090	0.60	−0.165	0.155	0.581	8.967
	0.3	0.000	0.003	0.032	0.083	0.90	0.314	0.215	0.593	7.506
	0.4	0.015	0.005	0.061	0.071	1.20	0.509	0.219	1.202	7.375
	0.5	−0.022	0.003	0.015	0.059	1.33	0.163	0.200	0.410	7.105
4c						0.00	0.025	0.010	−0.051	1.485
						0.30	−0.268	0.010	−0.351	2.085
						0.60	−0.565	0.010	−0.658	2.868
						0.90	−0.893	0.011	−0.918	3.852
						1.20	−1.133	0.037	−1.094	4.562
						1.33	−1.235	0.013	−1.284	4.430

1. Scenario 1: In the presence of regular confounding by ${Z}_{i}$ (addressed by direct adjustment in multilevel analyses and adjustment for aggregate ${Z}_{j}$ in the ecological analyses) all continuous and binary outcome models were free of bias (Fig. 3A and B). A key difference between multilevel and ecological analyses for either continuous or binary outcomes was greater heterogeneity among the ecological estimates (Table 2).
2. Scenario 2: In the presence of latent confounding by ${L}_{i}$ (addressed by adjusting for cluster size as its surrogate), all analyses suffered bias (Fig. 3C and D). Multilevel analyses suffered residual confounding bias (i.e., due to imperfect adjustment for cluster size) while ecological analyses suffered aggregation bias that was larger than residual confounding bias (Table 2). The ecological analyses also revealed a nonlinear degree of bias with respect to the specified causal effect (${\rho }_{7}$) for binary outcomes.
3. Scenario 3: In the presence of unrelated regular and latent confounding (by ${Z}_{i}$ and ${L}_{i}$, respectively), results were similar to Scenario 2 for continuous outcomes (Fig. 3E), but different for binary outcomes (Fig. 3F). The combination of regular and latent confounding yielded different biasing influences for binary outcomes due to aggregation and link-function transformation of the binary outcome. Ecological analyses consistently demonstrated larger estimate heterogeneity than multilevel analyses (Table 2). Both the multilevel and ecological analyses revealed a nonlinear degree of bias with respect to the specified causal effect (${\rho }_{7}$) for binary outcomes.
4. Scenario 4: In the presence of causally related regular and latent confounding (by ${Z}_{i}$ and ${L}_{i}$, respectively), the nature of biases altered for both continuous and binary outcomes. Multilevel analyses still fared better than the ecological analyses, with the latter more heterogeneous (Table 2).

- 4a. When the binary outcome was rare, both multilevel and ecological analyses were affected, with estimate heterogeneity for ecological analyses increasing substantially (Fig. 4a, Table 2).
- 4b. For continuous outcomes, binary latent confounding had modest biasing impact on both analyses but more so for the ecological analyses (Fig. 4b, Table 2).
- 4c. For binary outcomes combined with binary latent confounding some modest simulation bias was detected (see Appendix Section "Introduction") and estimate heterogeneity for the ecological analyses increased for larger effect sizes (Fig. 4c, Table 2).

Results from Scenario 1 indicate that if all variables are multivariate normal, point estimates of both multilevel and ecological analyses suffer only residual confounding bias. However, model heterogeneity is considerable for the ecological analyses (Table 2). Within a single study, sampling variation may lead to small errors for the multilevel analyses but substantial errors for ecological analyses. In Scenario 2, Model 2 was the least biased of all ecological analyses, but estimate heterogeneity was large (Table 2); analysis of any one sample will therefore be very uncertain. Scenario 3 indicated that multiple, unrelated, types of individual-level confounding exacerbated bias for binary outcomes, even though this is not implicated by the DAG for single-level analyses. Aggregation and the link-function transformation thus made a difference to the ecological analyses for binary individual-level outcomes. Scenario 4 indicated that multiple, causally related types of individual-level confounding exacerbate bias (again, not implicated by the DAG for single-level analyses) and this occurs for both continuous and binary individual-level outcomes. Bias among the ecological analyses of a binary individual-level outcome changed from Scenario 3, revealing sensitivities to the individual-level causal structures among confounders. The extended analyses of Scenario 4 demonstrated that multilevel analyses remain the most robust (Fig. 4a, Table 2). Binary confounders elevated bias for both continuous and binary outcomes for both multilevel and ecological analyses (Fig. 4b & c, Table 2). Overall, multilevel analyses consistently fared better than ecological analyses in terms of bias and errors with model estimates due to sample heterogeneity combined with aggregation.

## Discussion

Our first goal was to hypothesise a data generating mechanism (DGM) that describes a population in which research might be undertaken where interest lies in reliably estimating a population average causal effect of a causal phenomenon at the individual level. We explored circumstances relevant to studying non-communicable diseases for causal impacts affecting individuals in the presence of individual-level confounding that is causally linked to cluster size (Fig. 1). Our second goal was to simulate hierarchical data for this DGM, requiring a novel algorithm described in Fig. 2 to ensure we encapsulate cross-level causal relationships. This demonstrated how a multilevel path diagram may inform hierarchical data simulation with specified cross-level causal structure. Our final goal was to estimate the causal phenomenon at the individual level using multilevel and ecological analyses, evaluating the utility and robustness of each approach in obtaining the unbiased magnitude of simulated causal effects. Our results highlight the superiority of multilevel analyses over ecological analyses and illustrate the imperative of having individual-level data to investigate individual-level causal phenomena.

### Principal findings

Ecological analyses of causally structured hierarchical data revealed that cluster estimates of individual-level causal effects were more heterogeneous and less robust than multilevel analyses. Unobserved confounding at the individual level can be adjusted for in ecological analyses using cluster-level surrogates obtained either as aggregated summaries or causally related cluster variables, but this is less effective than adjusting for cluster-level surrogates in a multilevel analysis. Residual confounding always occurs but is less severe for multilevel analyses, which produce more homogeneous model estimates. Ecological analyses suffered aggregation bias due to engaging with variables purely at the cluster level, exacerbated by outcome link-function transformations for binary outcomes.

We simulated a known causal relationship between individual-level variables and cluster-size because the latter is often treated as though it ‘confounds’ (or, more likely, is a surrogate of unobserved confounders), even though the basis of this is rarely stated explicitly in terms of a priori causal origins. It would be helpful for researchers to be clearer in the future as to what cluster size represents in studies that adjust for it. Without undertaking causally structured simulations, there is no way of being sure that adjustment is either justified (as per an appropriate DAG) or indeed has the desired effect.

Given the assumptions adopted and ecological models evaluated, ecological analyses suffered estimate heterogeneity that overwhelmed residual confounding bias, and there are no obvious fixes to these issues. For binary outcomes, the simplest ecological analysis (Model 2, Table 1) did as well as the multilevel analysis (Model 1, Table 1) only when averaged over $1000$ repeated simulations, while sample heterogeneity meant that for any single dataset the ecological analysis is less favourable. Heterogeneity for the ecological analyses was exacerbated by the skewed distribution of aggregated counts for binary confounders.

For populations in which we seek to investigate non-communicable diseases and evaluate causal effects on individuals, individual-level data are needed. Each level of a natural hierarchy may be viewed through a causal lens, with each level encoded separately in a DAG, contributing to a multilevel causal diagram, where cross-level causal effects are captured via the algorithm outlined in Fig. 2.

### Limitations & improvements

There are several limitations to our study. First, we considered one DGM to reflect population homogeneity with no between-person interference, emulating the study of non-communicable diseases. These were deliberate simplifications; other DGMs likely reveal different patterns of bias, but the implications for most ecological analyses are clear. Alternative DGMs must be explored, e.g., where clusters are not from the same homogeneous population and/or between-person interactions arise, to understand the implications for the evaluation of these situations. The principles outlined in this study for data simulation extend to any real-world hierarchical context, though it is necessary to navigate DAG construction carefully after first obtaining a good grasp of the underlying data generating processes at work. This requires expertise in drawing DAGs – some specific considerations are outlined in Section "Important principles underpinning the development of causal graphs" of the Appendix.

Another limitation was to consider just one regular and one latent confounder. This nevertheless sufficed to show that multiple individual-level confounders can ‘interact’ to exacerbate ecological biases – where confounders are unrelated to each other, this may impact only binary outcomes, but where confounders are causally related, continuous outcomes are likely affected also. Confounders were also mostly multivariate normal, as focus was on differences in *outcome* distribution, but the extended Scenario 4 simulations demonstrated that binary latent confounding could exacerbate bias for the ecological analyses. More simulations might explore multiple binary confounders (i.e., for both ${Z}_{i}$ and ${L}_{i}$), but these are unlikely to show that ecological analyses will perform any better.

We also did not consider an exhaustive range of possible path coefficients, levels of confounding were modest, and we did not vary the complexity of simulations to accommodate features such other outcomes distributions, or complex parametrisations involving nonlinear relationships and/or covariate interactions. Scope for additional complexities is infinite, and we settled on a relatively straightforward representation of what is plausible and relevant for research in population health investigations into non-communicable diseases.

For computational reasons we made pragmatic choices in simulating a total population (${\varvec{N}}$) of only $100 000$ individuals with an arbitrarily $100$ clusters (${\varvec{C}}$). While headline messages would be similar, exact findings would differ had the ratio of ${\varvec{N}}$ to ${\varvec{C}}$ been different – i.e., MAUP previously described. Exploring MAUP was not our focus, but methods for its investigation are now more accessible using the algorithm described in Fig. 2.

Finally, we had to examine our simulated datasets for potential problems, and this is indicative of just how challenging it is to simulate causally structured data (even single-level data, as that was where we started). There is a paucity of solutions to this problem, despite emergence of several software packages that purportedly generate data of a prespecified causal nature. No software presently makes this easy for a mix of distributions within single-level data, let alone causally structured multilevel data. This gap in our toolkit limits our research capabilities and provides an ongoing challenge.

### Implications for ongoing population research

Practical implications of our work are: 1) simulations of causally structured hierarchical data are imperative for the evaluation of efficacy of methods within population research for all possible population data generating mechanisms; 2) methods reliant on synthetic data, such as ABMs, must be assured that synthetic data reflect all important causal structures, including cross-level effects; and 3) methodological issues with hierarchical data, such as MAUP, must be better understood – issues that are all readily examined using the foundation work of this study.

When seeking causal understanding in population research, adjustment for confounding (either directly or via surrogate measures) is vital for reliable and robust research insights. Many studies consider some form of adjustment for cluster size, though rarely is this justified. It might be that cluster size directly influences individual-level variables, or cluster size acts as a surrogate for such influences – for instance, acting in tandem with geographical size, as surrogate measures for air quality within clusters affected by daily transport usage and consequent air pollution that can impact respiratory and cardiovascular health. Perhaps no causal process is envisaged but heterogeneity across clusters is observed, invoking adjustment for cluster size to improve precision of within-cluster estimates.

If there are causal processes operating that give rise to cross-level influences related to cluster size, there are different ways to accommodate this (see Appendix, Section "Methods"). Creation of ratio variables by dividing through by cluster size does not remove its direct or surrogate confounding influences; such strategies have a severely deleterious effect on model estimates. The same is true for adjusting for the reciprocal of cluster size. The benefit of including an offset of the logarithm of cluster size in Poisson models for clustered counts – common in epidemiology [51] – has not been justified and our simulations show no benefit but only *increased* aggregation bias. It is unlikely that a log-size-offset could benefit robust causal inquiry for other DGMs – such insight to the limitations of a common analytical practice demonstrates the importance of causally structured simulations to evaluate the methods we use routinely.

Without a priori knowledge of causal structures among individual-level confounders and cluster-level surrogates, robust causal estimation of individual-level relationships is impossible – yet this is what is often wanted. Where ecological analyses are the only option, it is best to adjust directly for the best surrogate of all individual-level confounders, but this may yield enormously unreliable estimates in many instances. Detailed individual-level information is often hard to obtain due to data protection legislation that limits access to and use of personalised data [52]. Centralised population-based registers are rarely available to allow for a deterministic linkage of records across multiple domains such as sociodemographic characteristics and health. This gap in the availability of data has led to either ecological analyses or the creation of synthetic hierarchically structured data – the latter is increasingly used within public health and several other research domains.

The *Systems Science in Public Health and Health Economics Research* (SIPHER) consortium [53] has generated a synthetic population dataset for population health research across Great Britain [54], based on simulated annealing [55]. This spatial microsimulation approach optimises all aggregate-level patterns across small geographical areas but currently does not preserve causal structures within all levels or between levels of a natural hierarchy. Despite its advantage of being available for all Great Britain at a granular geographical resolution, the creation of SIPHER’s synthetic population does not embrace causal thinking and/or reflect a causal data generating processes. To our knowledge, no current simulation practice achieves this for complex hierarchical data structures. This is problematic where marginal correlations indicate associations between variables within and between levels of a data hierarchy and these relationships might be causal. Accounting for this is vital when relying on simulated data to conduct investigations using microsimulations or ABMs [53]. If known or hypothesised causal structures do not drive data simulations, it is impossible to explore if subsequent causal effect estimation for synthetic data is robust. The properties of utilised synthetic data and their impact on results must therefore be evaluated extensively for different contexts for researchers to be confident that resulting analyses are robust.

Complex systems abound and these will always be difficult to unravel causally, but we see increasing attempts to study the whole – i.e., a whole systems approach [56] – yet to achieve this we must improve our causal methods. Qualitatively speaking, this was addressed in obesity research when the Foresight systems map was published in 2007 [57]. Yet, despite considerable momentum in applying a whole systems approach to obesity [58–60], studies remain limited to qualitatively understanding the system. The value of blending multiple methods from the systems toolkit has been illustrated – for example, ABMs and system dynamic modelling [61] – but to date no research has taken a meaningful and robust quantitative perspective, evidenced by the lack of causally structured individual-level synthetic data orientated to the Foresight map. The algorithm outlined in Fig. 2 to generate causally structured hierarchical data is a step towards creating data for such complex systems, and the development of hierarchical causal diagrams is imperative to inform how best to conduct robust complex analyses in such data.

## Conclusions

Separation of within- and between-person causal effects is a legitimate objective and can be achieved with minimal bias using multilevel models. When information on all confounders is available, causal analyses must be informed by appropriate hierarchical causal diagrams to indicate which variables are included for each focal relationship of interest. Analyses that evaluate marginal relationships at the uppermost level of a data hierarchy (i.e., ecological analyses) generally cannot provide robust estimates of causal effects impacting at the individual level. While it has been known for some time that ecological analyses invoke the ecological fallacy [62] – i.e., where attributing features of clusters to units within clusters may mislead [63] – this has not been evaluated previously in a causal framework. We now reveal how and to what extent residual and/or unadjusted confounding bias and aggregation bias arise in ecological analyses from a causal perspective. For contexts involving regular and latent confounding at the individual level, ecological analyses will suffer residual confounding and aggregation biases, while multilevel models will suffer only residual confounding bias, and this is generally less than the estimate heterogeneity encountered with ecological analyses.

Interest in understanding the myriad impacts of a complex and rapidly changing world has never been greater, yet our data science capabilities to obtain robust causal insights of population systems – whether local, national, or international – remain woefully inadequate. We need more robust quantitative capabilities to help decision-makers arrive at robust evidence-based decisions. Our analyses must be able to assess accurately the causal processes impacting humans at the individual level. To do this, we must understand our capabilities to evaluate causality in complex hierarchical systems.

Within population research, the role of cluster size and the interpretation of cross-level associations with individual-level relationships must be carefully considered in a causal framework, which warrants the creation of causal diagrams for all levels of a data hierarchy. While some hierarchies are arbitrary, some matter – hence MAUP – realising and respecting this is essential. We should abandon the naïve approach of correlational analyses and only perform robust causal inquiries using state-of-the-art causal inference methods, even though these are still under development for hierarchical data structures. Many challenges remain, but this study starts to address the methods gap.

## Figures and Tables

**Fig. 5:**
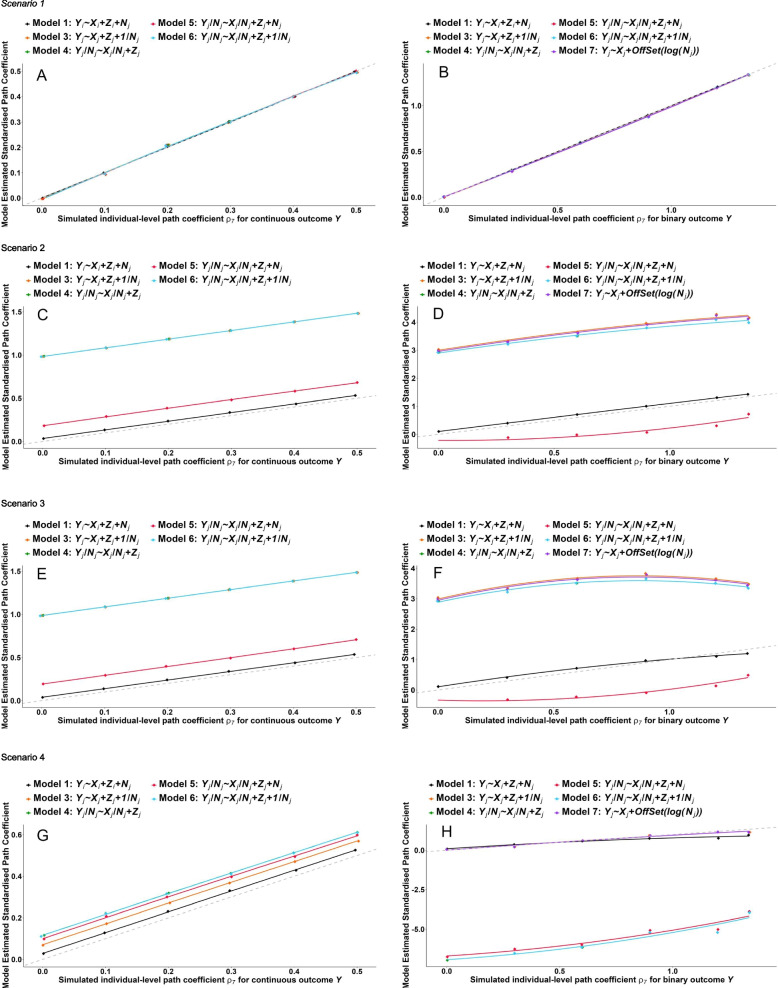
Summary of the multilevel and additional ecological analyses of simulated data considered (Table 1, Models 3 to 7) for all four scenarios for continuous (charts **A**, **C**, **E**, **G**) and binary outcomes (charts **B**, **D**, **F**, **H**) – the diamond shaped plots are median estimates (y-axis) plotted against individual-level simulated ‘true’ effect sizes (x-axis); the dotted grey line indicates perfect agreement between simulated and estimated effect sizes; continuous lines are fitted lines to the median estimates

## Data Availability

The R-code (to generate and analyse the data) will be available on the GitHub repository and published after publication.

## Appendix

### Details of the simulation procedure

To simulate multivariate normal data, the impliedCovarianceMatrix routine in the R package dagitty was used with the prespecified path coefficients of each level 1 DAG Scenario. To simulate a mixture of multivariate distributions we used the GenData routine in R developed by Ruscio and Kaczetow [49], which benefits from prespecifying the number of latent factors used to ensure reliable simulations. The number of factors to consider is between one and the total number of variables simulated less one. The GenData algorithm was initially tuned over 500 repeats, and then over 1000, and finally 5000 repeats, to explore consistency. This process was computationally intensive and on a 32-core workstation took nearly 4 days. The final simulations and analyses of all datasets took a further 6 days.

#### GenData tuning

The tuning involved first using the impliedCovarianceMatrix routine in R package dagitty to obtain the *exact* covariance of each parameterised DAG, where ‘exact’ means that simulated multivariate normal data for the DAG were equivalent to that obtained using the mvrnorm routine in R with the option empirical = TRUE – this removes variations due to random sampling and the covariance of the simulated data was thus exactly that implied by the parameterised level 1 DAG. This is not realistic for real-world situations but useful to evaluate the performance of each simulation. When simulating data for continuous outcome scenarios we used the option empirical = FALSE to deliberately invoke random sampling variation. The GenData routine is never exact and always simulates multivariate data with sample variation. When simulating multiple datasets using GenData for each DAG-specified covariance structure we contrasted the observed covariance structure to that obtained in R package dagitty for empirical = TRUE and averaged deviations for each simulated dataset to assess by how much each simulated dataset deviated from the targeted covariance structure in five ways: 1) the number of simulation attempts that failed to return data, i.e., GenData failed to converge – this occurred occasionally for some specifications or for all attempts at a given number of latent factors; 2) root-mean-square (RMS) of all contrasts of pairwise bivariate correlations between simulated and targeted standardised covariance matrices such that dividing through RMS values for each of the factor options by the smallest RMS ensures the optimal number of factors has an RMS of 1.0 and all other factor options had RMS values greater >1.0; 3) RMS of pairwise correlations involving only outcome *Y*, as this is the outcome in the analyses; 4) standard deviation (SD) of all pairwise bivariate deviations between simulated and targeted standardised covariance matrices since large SDs could indicate volatility and potential bias in some datasets; and 5) the time taken to execute repeated simulations for a specific number of latent factors, as this helps differentiate quicker options if there are multiple options available.

Table A3 summarises the tuning process for the first half of Scenario 1 and the second half of Scenario 4 for the main simulations. The size of bias increased with increasing constraints imposed by the DAG for increasing path coefficient values. For many configurations there was a clear first choice for the number of factors, sometimes a second choice. First choice options delivered reasonable simulations. Simulating data for the extensions to Scenario 4 revealed that some did not converge – this did not prohibit successful tuning. Simulation challenges increase for non-Gaussian variables that are ancestors to other variables. Consider the simplified DAG for sex and body weight, for instance, where the former causes the latter (ignoring all other factors). Using the covariance of sex and weight, GenData starts with two variable distributions: binomial with specified male-female proportions and a Gaussian with mean and SD (this is for all males and all females combined). Simulated data are found by suitable mixing of both distributions to obtain the target covariance, yielding data in which the first two moments (i.e., mean and variance, plus variable covariances) are obtained. With correctly derived first two moments, first-order fixed effects and associated standard errors will be robust in the analyses of the simulated data. In this example, it is anticipated that population weight is bimodal – a mixture of two Gaussians; one for each sex. Simulating data that are generated longitudinally (i.e., sex precedes weight) from a cross-sectional covariance (i.e., agnostic to time) is less likely to yield simulated data with perfect correspondence to real-world data (i.e., where weight is a clear mixture of two Gaussians). This is why simulated annealing, currently used by SIPHER, is superior for capturing internal structure that evolves due to temporal data generation, though currently this approach does not ensure all within- and between-level causal structures are realised. With temporal dimension we refer to the assumption that margins seen in observed data can be understood as being caused by the temporal flow of causality (e.g., sex precedes weight). This temporal flow of causality is currently not accounted for in GenData. We thus need better methods of simulating complex causally structured data. For our study, GenData sufficed to generate data with causal structures, but future studies should seek tools that combine advantages of simulated annealing and DAG-informed simulation.

#### Additional internal validation checks of the simulated datasets

We also evaluated how well data were simulated by exploring the results of multilevel models where adjustment was made for both regular and latent confounding *at the individual level* (i.e., adjusting for $Z_{i}$ and $L_{i}$ explicitly). These multilevel estimates should be close to truth and by examining median values over repeated simulations when the causal effect is zero (i.e., $\rho_{7}=0$) identifies biases introduced by the simulation. Zero or negligible bias (<0.001) was detected for the main four binary outcome models whereas larger biases were detected for Scenario 4 extensions: 0.208 for 4a (low prevalence binary outcome); 0.049 for 4b (binary confounding, continuous outcome); and 0.111 for 4c (binary confounding, binary outcome) – indicating that GenData was not perfect. These biases determined when $\rho_{7}=0$ were subtracted from the estimates for path coefficients $\rho_{7}>0$ for the Scenario 4 extensions when results were summarised graphically in Fig. 4. While reasons for such biases remain unclear, these were modest, and headline conclusions were unaffected.

### Graphical results of ecological Models 3 to 7 versus multilevel Model 1

The main text focusses on the straightforward adjustment for cluster size in both the multilevel (Model 1, Table 1) and main ecological analyses (Model 2, Table 1). Here we detail additional ecological analyses, which included; Model 3 – adjustment for the inverse of cluster size, $1/N_{j};$ Model 4 – analysis of ratios, where $Y_{j}/N_{j}$ was examined in relation to $X_{j}/N_{j};$ Model 5 – analysis of ratios with the additional adjustment for cluster-size, $N_{j};$ Model 6 – analysis of ratios with additional adjustment for the inverse of cluster size,$1/N_{j}$; and for binary outcomes only, Model 7 – adjustment for the log-size-offset term $offset(logN)$, which has been recommended in epidemiology for clustered counts [51], though no formal rationale for this could be found. To illustrate the analytical differences for a more technical reader, we summarise the difference algebraically between the multilevel analysis and the ecological analysis (where *i* depicts the individual level and *j *the cluster level):

Multilevel: $$Y_{ij}=\beta_{0ij}+\beta_{1j}X_{ij}+\beta_{2ij}Z_{ij}+\beta_{3j}N_{j}+u_{oj}+e_{ij}$$ 

Ecological: $\hat{Y}_{j}=\hat\beta_{0j}+\hat\beta_{1j}\hat{X}_{j}+\hat\beta_{2j}\hat{Z}_{j}+\hat\beta_{3j}N_{j}+\epsilon_{j}$where $u_{0j}$ and $e_{ij}$ multilevel random effects at the cluster and individual levels, respectively, and $\epsilon_{j}$ is the ecological random effect at the cluster level; and the variable hat indicates an individual level variable that has been aggregated to the cluster level. It should be noted that for ratio analyses of continuous individual-level variables the aggregated variable is cluster mean (i.e., derived after dividing the aggregated cluster values by $N_{j}$), but since dividing a measure through by the source of its heterogeneity is common practice to ‘standardise’ that measure, we consider the situation where division by $N_{j}$ occurs twice for continuous outcome Models 4 to 6.

#### Results for ecological Models 3 to 7 versus multilevel Model 1

Figure A5 summarises the results for the additional ecological analyses Models 3 to 7 (Table 1). For Scenario 1, all continuous and binary outcome models were free of bias (Figures A5A and B). For Scenario 2, all analyses suffered bias (Figures A5C and D), with all ecological analyses suffering considerable aggregation bias. Results for Scenario 3 were similar to results Scenario 2 for continuous outcomes, but different for binary outcomes (Figures A5E and F). Results for Scenario 4 differed substantively for both continuous and binary outcomes, revealing the impact that causally linked multiple confounders can have on all analyses. The impacts of linked multiple confounding were particularly exacerbated for binary outcomes due to the combined influences of aggregation and link-function transformation of the outcome.

The creation of ratios by dividing the outcome by the cluster size had severely deleterious effects on model estimates, as too sometimes did adjusting for the reciprocal of cluster size. These results for ratio variables have implications for other ratio variables (e.g., body mass index, BMI) – if a variable is constructed to ‘standardise’ one measure (e.g., weight) with respect to another variable that yields heterogeneity (e.g., height), construction of ratios is *not* the same as, nor a viable alternative to, direct adjustment (as for genuine confounders). The assumption typically underpinning confounders is that they, not their reciprocal, are linearly or near-linearly related to the exposure and the outcome. While other parametric assumptions may be necessary (e.g., nonlinearity and interactions with other variables), at no point is it helpful to take variables that demonstrate heterogeneity and divide through them by causes of that heterogeneity [43]. A causal diagram can inform appropriate adjustment, with parametric considerations explored once the adjustment set is known.

The practice of including an offset of the logarithm of cluster size in Poisson models for clustered counts maybe often be used in epidemiology [51], but the benefits of this have not been made clear. One reason might stem from viewing clustered counts as rates, i.e., the frequency of individual-level binary outcomes within each cluster divided by cluster size. Mathematically deconstructing the log link used in Poisson modelling $[log(Y/N)=log(Y)-log(N)]$, we switch the outcome from a rate to a count by adding $log(N)$ to the linear predictor of model covariates. It is preferable to avoid analysing ratio variables, as this invokes several problems [64], but the notion that dividing through an outcome by a confounder to ‘standardise’ that outcome is misguided, evidenced by the ecological analyses of ratios. The correct way to accommodate outcome heterogeneity associated with cluster size is to treat it as a confounder, i.e., as part of the linear combination of model covariates, *not* by dividing through by it. Although use of a log-size-offset may seem mathematically intuitive, for our DGM, simulations demonstrate that it provided no benefit and even exacerbated bias, meaning that many existing studies in the literature may have provided biased findings.

### Practical application of the algorithm summarised in Figure 2

The principles underpinning application of the algorithm in Fig. 2 are that to ensure *within-level causal structure* is imposed for each level of a hierarchy, a causal graph must contain all variables for each level based on *a priori* knowledge or postulated theory of the data generating processes and causal phenomena at that level; a separate causal graph is necessary for each level at which causal structure is assumed. Each graph yields implied constraints for a covariance matrix of all variables at that level. Within the simulated annealing process (as used by SIPHER), a causal graph at each level yields marginal constraints to inform the simulated annealing. This differs from what is current practice only in that causal graphs are comprehensive (defining constraints for all variables) and define narrower bounds within which covariances can fluctuate due to sampling heterogeneity than might be assumed from an observed covariance matrix. In nonparametric causal diagrams, graph-implied covariance structure is one-to-many in both directions – i.e., for a given causal graph, there are many (sometimes infinite) plausible covariance structures, and for any given covariance structure there are many (but finite, due to the number of variables) plausible causal graphs. A causal graph plus observed covariance is more restrictive if the graph has missing arcs, since this reflects stronger assumptions. The temporal order of variables plus absent arcs thus provides greater constraint than the observed covariance structure alone. This does not, however, capture cross-level causal relationships.

To ensure *between-level causal structure* is captured, a latent ‘surrogate variable’ is needed at the lowest level at which the cross-level causal process originates (e.g., $N_{i}$ at level 1 in our study); this latent variable then propagates the cross-level relationship upwards through the hierarchy via our algorithm to generate cross-level dependencies (e.g., between lower-level variables $X_{i}$ and $Y_{i}$ and upper-level cluster size, $N_{j}$, in our study). We considered two levels, but the algorithm, in principle, extends for as many levels and as many cross-level effects as needed. With careful consideration of all lower-level latent origin variables (e.g., $N_{i}$), it is feasible to generate multiple cross-level dependencies, omitting intermediate levels if required. This requires an appreciation of how all effects relate to each other at each level, requiring links between sets of latent origin variables to ensure clusters are appropriately generated. This is feasible for all hierarchical data structures, such as cross-classified and/or multiple membership data.

As the algorithm is not simulation-tool dependent it can be incorporated within any process that implements appropriate synthetic data generation, providing this is informed by the hierarchical causal diagram.

### Important principles underpinning the development of causal graphs

A causal diagram, such as a DAG, requires researchers to postulate which variables are relevant for the research question(s) in hand, and whether these are directly measurable or not – where not directly measurable, surrogate variables that indirectly measure missing variables should be considered. The drawing of reliable causal graphs is hard and often requires multiple iterations and persistent team effort, ideally involving a mix of causal inference and domain knowledge experts. Although initially quite challenging, practice improves this skill. Many snippets of advice exist in the literature (see for instance: [18, 65]), but these are not exhaustive.

Here, we summarise the key issues briefly: 1) place variables in temporal order of ‘crystallisation’ (i.e., the time when measures stabilise in capturing what is being sought – for instance, some measures, such as blood pressure, are volatile and tend to yield ‘in-the-moment’ assessments, whereas other measures, such as hypertension, evolve slowly and only attain some critical threshold over a period of time); for variables that crystalise contemporaneously, these are placed at the same times in the temporal flow of the diagram and have no arcs directly between them but may have common unobserved (i.e., latent) variables as common ancestors (to invoke non-zero bivariate correlations between them, if warranted) – example variables that might be placed contemporaneously are *age*, *sex*, and *ethnicity* (without latent common ancestors, unless within a context where these are warranted); 2) draw arcs by assuming forward ‘saturation’ (i.e., each variable in the causal diagram may cause all future variables) and only remove an arc if there is strong evidence to justify this (mechanistically or from robust evidence in the literature), since the absence of an arc is a stronger assumption than the presence of an arc.

Reliance on the literature can be precarious since many observational research studies fail to embrace contemporary causal inference methodology and might be deemed speculative, not robust. It is however possible to evaluate consistency between the causal diagram and available data [42]. Missing vital variables – whether this be the omission of confounders, the failure to understand complex causal structure among multiple confounders, leading to the risk of M-bias [39], or some other misspecification of the causal graph – will likely lead to biased estimates of the data when analysed, the extent of which is unknown. For missing confounders, estimates will suffer unmodelled confounding bias, while for M-bias, estimates may be adjusted in the wrong direction (i.e., become more, not less biased). For any misspecification of the causal diagram, there will be consequences, but no more so than where data are simulated using approaches that fail to incorporate vital causal structures. Causal diagrams encode all data generating assumptions explicitly, which makes them open to scrutiny, discussion, and continual improvement. This is why it is imperative to embrace causal thinking and appropriate methods in observational research, whenever we seek causal insights.

When seeking to develop a multilevel causal diagram, it is necessary to remain cognizant of how to deal with fully determined variables – this applies both within each level and between levels, with aggregated variables determined by lower-level variables. Development of a causal diagram for each level might proceed differently according to context. For the present study, we began with a DAG at the individual level and developed the dataset to contain cluster-level variables through aggregation, whereas if seeking to emulate the population heterogeneity context (i.e., where clusters differ substantially, consistent with being distinct countries), early considerations of a multilevel causal diagram indicate this is better generated starting with the cluster-level DAG. Each different hierarchical data generating structure must be considered on a case-by-case basis – it is infeasible to illustrate all potential contexts and associated strategies to develop all possible multilevel causal diagrams within a single study; we did not embark upon such a task as the number of unique possibilities have not yet been numerated.

Embarking on the development of causal diagrams for observational research makes scientific enquiry more reproducible, with refinements possible in a more open forum. In observational research, causal origins are often nebulous, typically complex, and reliable assessment of cause and effect is challenging due to limited availability of all relevant information and associated data. It has always been thus. Only by being explicit in our assumptions, in ways that causal diagrams demand, can we hope to improve the robustness of observational research.

**Table 3:** A snapshot summary of the GenData tuning process for the first and last three subsets of the four main Scenarios examined. Bolded cells represent options that were optimal or near optimal within each scenario for each specified path coefficient ($\rho_{7}$)

**Scenario**	$\rho_{7}$	**n Latent Factors**	**Failed Simulations**	**Total Bias**	**Y-Bias**	**SD**	**Time**	**Overall Relative Bias**	**Scenario Relative Bias**	**Scenario Relative Y-Bias**	**Scenario Relative SD**	**Scenario Relative Time**	**Selected Number of Factors**
1	0	1	0	0.0069	0.0039	0.0043	5.7333	1.00	1.49	1.57	**1.00**	**1.07**	4
1	0	2	0	0.0053	0.0038	0.0061	5.3740	1.00	1.15	1.52	1.40	**1.00**	
1	0	3	0	0.0049	0.0031	0.0074	6.7069	1.00	**1.07**	1.25	1.70	1.25	
1	0	4	0	0.0046	0.0025	0.0074	6.7015	1.00	**1.00**	**1.00**	1.70	1.25	
1	0.1	1	0	0.0060	0.0026	0.0037	5.2125	1.2	1.69	1.35	**1.00**	**1.00**	2
1	0.1	2	0	0.0036	0.0019	0.0042	5.1979	1.2	**1.01**	**1.00**	1.11	**1.00**	
1	0.1	3	0	0.0035	0.0020	0.0072	6.4742	1.2	**1.00**	**1.02**	1.92	1.25	
1	0.1	4	0	0.0047	0.0026	0.0075	6.5846	1.2	1.33	1.36	2.01	1.27	
1	0.2	1	0	0.0074	0.0045	0.0045	4.8194	1.3	1.60	1.76	**1.00**	**1.00**	4
1	0.2	2	0	0.0057	0.0048	0.0048	5.1677	1.3	1.24	1.87	**1.06**	**1.07**	
1	0.2	3	0	0.0062	0.0049	0.0065	5.7489	1.3	1.33	1.92	1.44	1.19	
1	0.2	4	0	0.0046	0.0026	0.0072	6.7903	1.3	**1.00**	**1.00**	1.59	1.41	
4	0.3	1	0	0.4025	0.3731	0.0062	2.3615	6265	1.20	1.12	**1.00**	**1.00**	4
4	0.3	2	0	0.3470	0.3384	0.0065	5.4388	6265	**1.04**	**1.02**	**1.04**	**2.30**	
4	0.3	3	0	0.3628	0.3625	0.0093	2.8618	6265	**1.08**	**1.09**	1.51	1.21	
4	0.3	4	0	0.3348	0.3328	0.0063	2.8608	6265	**1.00**	**1.00**	**1.02**	1.21	
4	0.4	1	0	0.5360	0.5014	0.0062	2.3714	8735	1.15	1.08	**1.00**	1.00	4
4	0.4	2	0	0.4757	0.4696	0.0065	3.2807	8735	**1.02**	**1.01**	**1.04**	1.38	
4	0.4	3	0	0.4668	0.4634	0.0067	2.9556	8735	**1.00**	**1.00**	**1.07**	1.25	
4	0.4	4	0	0.4815	0.4747	0.0066	2.8707	8735	**1.03**	**1.02**	**1.06**	1.21	
4	0.5	1	0	0.6713	0.6306	0.0064	2.3714	11388	**1.10**	1.05	**1.00**	**1.00**	4
4	0.5	2	0	0.6086	0.6010	0.0159	3.8088	11388	**1.00**	**1.00**	2.48	1.61	
4	0.5	3	0	0.6183	0.6065	0.0096	3.0631	11388	**1.02**	**1.01**	1.50	1.29	
4	0.5	4	0	0.6319	0.6185	0.0065	2.9399	11388	**1.04**	**1.03**	**1.02**	1.24	

## References

[1] Europe UNEC. Register-based statistics in the Nordic countries: review of best practices with focus on population and social statistics. 2007.

[2] Dehghani M, Tumer M. A research on effectiveness of Facebook advertising on enhancing purchase intention of consumers. Comput Hum Behav. 2015;49:597–600.

[3] Tate RL. Cross-level interaction in multilevel models. J Appl Behav Sci. 1985;21:221–34.

[4] Goldstein H, Browne W, Rasbash J. Multilevel modelling of medical data. Stat Med. 2002;21:3291–315. 12375305 10.1002/sim.1264

[5] Guthrie B, Donnan PT, Murphy DJ, Makubate B, Dreischulte T. Bad apples or spoiled barrels? Multilevel modelling analysis of variation in high-risk prescribing in Scotland between general practitioners and between the practices they work in. BMJ Open. 2015;5: e008270. 26546137 10.1136/bmjopen-2015-008270 PMC4636636

[6] Curran PJ. Have Multilevel Models Been Structural Equation Models All Along? Multivar Behav Res. 2003;38:529–69. 10.1207/s15327906mbr3804_5 26777445

[7] Skrondal A, Rabe-Hesketh S. Generalized Latent Variable Modeling: Multilevel, Longitudinal, and Structural Equation Models. London: Chapman & Hall; 2004.

[8] Goldstein H, Carpenter JR, Browne WJ. Fitting multilevel multivariate models with missing data in responses and covariates that may include interactions and non-linear terms. J R Stat Soc Ser A. 2014;177:553–64.

[9] Arnold KF, Davies V, de Kamps M, Tennant PW, Mbotwa J, Gilthorpe MS. Reflections on modern methods: generalized linear models for prognosis and intervention—theory, practice and implications for machine learning. Int J Epidemiol. 2020;49:2074–82. 10.1093/ije/dyaa049 PMC7825942 32380551

[10] Huitfeldt A. Is caviar a risk factor for being a millionaire? BMJ. 2016;355: i6536. 27940434 10.1136/bmj.i6536

[11] Heppenstall AJ, Crooks AT, See LM, Batty M. Agent-based models of geographical systems. Springer Science & Business Media; 2011.

[12] Arnold KF. Statistical and simulation-based modelling approaches for causal inference in longitudinal data: Integrating counterfactual thinking into established methods for longitudinal data analysis (Doctoral dissertation, University of Leeds). PhD Thesis. University of Leeds; 2020.

[13] Gilthorpe MS, Frydenberg M, Cheng Y, Baelum V. Modelling count data with excessive zeros: the need for class prediction in zero-inflated models and the issue of data generation in choosing between zero-inflated and generic mixture models for dental caries data. Stat Med. 2009;28:3539–53. 19902494 10.1002/sim.3699

[14] Rubin DB. Causal inference using potential outcomes: Design, modeling, decisions. J Am Stat Assoc. 2005;100:322–31.

[15] Pearl J. The algorithmization of counterfactuals. Ann Math Artif Intell. 2011;61:29.

[16] Pearl J. Causal diagrams for empirical research. Biometrika. 1995;82:669–88.

[17] Greenland S, Pearl J, Robins JM. others. Causal diagrams for epidemiologic research Epidemiology. 1999;10:37–48. 9888278

[18] Tennant PWG, Murray EJ, Arnold KF, Berrie L, Fox MP, Gadd SC, et al. Use of directed acyclic graphs (DAGs) to identify confounders in applied health research: review and recommendations. Int J Epidemiol. 2021;50:620–32. 33330936 10.1093/ije/dyaa213 PMC8128477

[19] Naimi AI, Cole SR, Kennedy EH. An introduction to g methods. Int J Epidemiol. 2017;46:756–62. 28039382 10.1093/ije/dyw323 PMC6074945

[20] Shardell M, Ferrucci L. Joint mixed-effects models for causal inference with longitudinal data. Stat Med. 2018;37:829–46. 29205454 10.1002/sim.7567 PMC5799019

[21] Xu Y, Kim J, Hummers LK, Shah AA, Zeger S. Causal Inference using Multivariate Generalized Linear Mixed-Effects Models with Longitudinal Data. arXiv preprint arXiv:230302201. 2023. 10.1093/biomtc/ujae100 PMC11422711 39319549

[22] Bijlsma MJ, Wilson B. Modelling the socio-economic determinants of fertility: a mediation analysis using the parametric g-formula. J R Stat Soc Ser A Stat Soc. 2020;183:493–513.

[23] Hale JM, Bijlsma MJ, Lorenti A. Does postponing retirement affect cognitive function? A counterfactual experiment to disentangle life course risk factors. SSM-Population Health. 2021;15: 100855. 34258375 10.1016/j.ssmph.2021.100855 PMC8255239

[24] VanderWeele TJ. Mediation analysis: a practitioner’s guide. Annu Rev Public Health. 2016;37:17–32. 26653405 10.1146/annurev-publhealth-032315-021402

[25] Legendre P, Fortin MJ. Spatial pattern and ecological analysis. Vegetatio. 1989;80:107–38.

[26] Gehlke CE, Biehl K. Certain effects of grouping upon the size of the correlation coefficient in census tract material. J Am Stat Assoc. 1934;29:169–70.

[27] Coombes MG, Dixon JS, Goddard J, Openshaw S, Taylor PJ. Daily urban systems in Britain: from theory to practice. Environ Plan A. 1979;11:565–74.

[28] Openshaw S. The modifiable areal unit problem. Concepts and techniques in modern geography. 1984.

[29] Fotheringham AS, Wong DW. The modifiable areal unit problem in multivariate statistical analysis. Environ Plan A. 1991;23:1025–44.

[30] Kwan M-P. From place-based to people-based exposure measures. Soc Sci Med. 2009;69:1311–3. 19665828 10.1016/j.socscimed.2009.07.013

[31] Nelson JK, Brewer CA. Evaluating data stability in aggregation structures across spatial scales: revisiting the modifiable areal unit problem. Cartogr Geogr Inf Sci. 2017;44:35–50.

[32] Wang Y, Di Q. Modifiable areal unit problem and environmental factors of COVID-19 outbreak. Sci Total Environ. 2020;740: 139984. 32534259 10.1016/j.scitotenv.2020.139984 PMC7274979

[33] Dusek T. The modifiable areal unit problem in regional economics. 2005.

[34] Puga D. The magnitude and causes of agglomeration economies. J Reg Sci. 2010;50:203–19.

[35] Pietrzak MB. Modifiable Areal Unit Problem: the issue of determining the relationship between microparameters and a macroparameter. Oeconomia Copernicana. 2019;10:393–417.

[36] Westreich D, Greenland S. The table 2 fallacy: presenting and interpreting confounder and modifier coefficients. Am J Epidemiol. 2013;177:292–8. 23371353 10.1093/aje/kws412 PMC3626058

[37] Tu YK, West R, Ellison GT, Gilthorpe MS. Why evidence for the fetal origins of adult disease might be a statistical artifact: the “reversal paradox” for the relation between birth weight and blood pressure in later life. Am J Epidemiol. 2005;161:27–32. 15615910 10.1093/aje/kwi002

[38] Cole SR, Platt RW, Schisterman EF, Chu H, Westreich D, Richardson D, et al. Illustrating bias due to conditioning on a collider. Int J Epidemiol. 2010;39:417–20. 19926667 10.1093/ije/dyp334 PMC2846442

[39] Ding P, Miratrix LW. To adjust or not to adjust? Sensitivity analysis of M-bias and butterfly-bias. Journal of Causal Inference. 2015;3:41–57.

[40] Pearl J, Glymour MM, Jewell NP. Causal Inference in Statistics: A Primer. London: Wiley; 2016.

[41] Mbotwa JL, de Kamps M, Baxter PD, Ellison GT, Gilthorpe MS. Latent class regression improves the predictive acuity and clinical utility of survival prognostication amongst chronic heart failure patients. PLoS ONE. 2021;16: e0243674. 33961630 10.1371/journal.pone.0243674 PMC8104399

[42] Textor J, van der Zander B, Gilthorpe MS, Liśkiewicz M, Ellison GT. Robust causal inference using directed acyclic graphs: the R package ‘dagitty.’ Int J Epidemiol. 2016;45:1887–94. 28089956 10.1093/ije/dyw341

[43] Berrie L, Arnold KF, Tomova GD, Gilthorpe MS, Tennant PW. Depicting deterministic variables within directed acyclic graphs (DAGs): An aid for identifying and interpreting causal effects involving tautological associations, compositional data, and composite variables. Epidemiology. 2024;24:kwae153. 10.1093/aje/kwae153 PMC11815499 38918044

[44] Ogburn EL, VanderWeele TJ. Causal diagrams for interference. 2014.

[45] Wright S. Correlation and causation. 1921.

[46] Wright S. The method of path coefficients. Ann Math Stat. 1934;5:161–215.

[47] WHO. Rare diseases. https://www.rarediseasesinternational.org/description-for-rd/.

[48] King G, Zeng L. Logistic regression in rare events data. Polit Anal. 2001;9:137–63.

[49] Ruscio J, Kaczetow W. Simulating multivariate nonnormal data using an iterative algorithm. Multivar Behav Res. 2008;43:355–81. 10.1080/00273170802285693 26741201

[50] R Core Team. R: A Language and Environment for Statistical Computing. Vienna, Austria: R Foundation for Statistical Computing; 2023.

[51] Breslow NE, Day NE. Statistical methods in cancer research volume II–the design and analysis of cohort studies. IARC Scientific Publications) Statistical Methods in Cancer Research. 1986;1:136–7. 3329634

[52] General Data Protection Regulation (GDPR). 2016. https://gdpr-info.eu/.

[53] SIPHER: Systems science In Public Health and Economics Research. https://www.gla.ac.uk/research/az/sipher/.

[54] Lomax N, Hoehn A, Heppenstall A, Purshouse R, Wu G, Zia K, Meier P. SIPHER Synthetic Population for Individuals in Great Britain, 2019-2021. [data collection]. University of Essex, Institute for Social and Economic Research, Office for National Statistics, [original data producer(s)]. University of Essex, Institute for Social and Economic Research; 2024. SN: 9277. 10.5255/UKDA-SN-9277-1.

[55] Harland K. Microsimulation Model user guide (flexible modelling framework). 2013.

[56] Höhn A, Stokes J, Pollack R, Boyd J, Del Cerro CC, Elsenbroich C, et al. Systems science methods in public health: what can they contribute to our understanding of and response to the cost-of-living crisis? J Epidemiol Community Health. 2023;77:610–6. 37328262 10.1136/jech-2023-220435 PMC10423532

[57] Vandenbroeck P, Goossens J, Clemens M. Foresight: tackling obesities: future choices-building the obesity system map. 2007.

[58] Public Health England. Whole systems approach to obesity. A guide to support local approaches to promoting a healthy weight. 2019.

[59] Allender S, Owen B, Kuhlberg J, Lowe J, Nagorcka-Smith P, Whelan J, et al. A Community Based Systems Diagram of Obesity Causes. PLoS ONE. 2015;10: e0129683. 26153893 10.1371/journal.pone.0129683 PMC4496094

[60] Waterlander WE, Luna Pinzon A, Verhoeff A, Den Hertog K, Altenburg T, Dijkstra C, et al. A system dynamics and participatory action research approach to promote healthy living and a healthy weight among 10–14-year-old adolescents in Amsterdam: The LIKE programme. Int J Environ Res Public Health. 2020;17:4928. 32650571 10.3390/ijerph17144928 PMC7400640

[61] Hennessy E, Economos CD, Hammond RA. Integrating complex systems methods to advance obesity prevention intervention research. Health Educ Behav. 2020;47:213–23. 32090653 10.1177/1090198119898649

[62] Robinson WS. Ecological Correlations and the Behavior of Individuals. Am Sociol Rev. 1950;15:351–7. 10.1093/ije/dyn357 19179346

[63] Schwartz S. The fallacy of the ecological fallacy: the potential misuse of a concept and the consequences. Am J Public Health. 1994;84:819–24. 8179055 10.2105/ajph.84.5.819 PMC1615039

[64] Tu Y-K, Law GR, Ellison GT, Gilthorpe MS. Ratio index variables or ANCOVA? Fisher’s cats revisited Pharm Stat. 2010;9:77–83. 19337988 10.1002/pst.377

[65] Digitale JC, Martin JN, Glymour MM. Tutorial on directed acyclic graphs. J Clin Epidemiol. 2022;142:264–7. 34371103 10.1016/j.jclinepi.2021.08.001 PMC8821727

